# Relationship between a Radiographic Grading Scale of Degenerative Lumbar Disease and Quality of Life

**DOI:** 10.1055/s-0044-1792115

**Published:** 2024-12-21

**Authors:** Réjelos Charles Aguiar Lira, Raphael de Rezende Pratali, Murilo Tavares Daher, Gabriel Henrique Pokorny, Rodrigo Augusto do Amaral, Carlos Fernando Pereira da Silva Herrero

**Affiliations:** 1 Faculdade de Medicina de Ribeirão Preto, Universidade de São Paulo, Ribeirão Preto, SP, Brasil; 2 Hospital do Servidor Público Estadual de São Paulo, São Paulo, SP, Brasil; 3 Grupo Brasileiro de Estudo de Coluna, São Paulo, SP, Brasil

**Keywords:** health-related quality of life, indicators of quality of life, intervertebral disc degeneration, osteoarthritis, spine, radiography

## Abstract

**Objective** To evaluate the correlation between a radiographic scale of lumbar degenerative disease and the Oswestry Disability Index (ODI).

**Methods** A cross-sectional study in which the ODI quality of life questionnaire and the radiographic parameters for the classification of lumbar degenerative disease into different grades were compared to try to establish a relationship between them.

**Results** The relationship between the radiographic parameters and quality of life indicators does not behave homogeneously, considering the different grades of the lumbar grading scale. Grade-2 lumbar degenerative disease showed a statistically significant relationship with the worsening of the ODI quality of life score.

**Conclusion** The lumbar degenerative disease grading scale used in the present study showed a relevant clinical potential, as it presented a significant relationship with the quality of life measured by the ODI score in part of the groups evaluated.

## Introduction

One of the leading causes of chronic low back pain is lumbar degenerative disease (LDD), which is characterized by a series of changes, especially in the intervertebral disc, called degenerative disc disease (DDD). The evolution of this process and the mechanisms by which such changes cause pain are still topics of discussion in the literature. 1 2 The characteristic radiographic findings of DDD include decreased disc space height, osteophytosis, and endplate irregularity, which may or may not be associated with deformities such as scoliosis, spondylolisthesis, or degenerative laterolisthesis. However, such findings in radiographic examinations of asymptomatic individuals are common, and the association between the described findings and symptomatology still needs to be fully established. 3

Another important cause of chronic low back pain is the disease group that falls into the scope of adult spinal deformity (ASD), 4 which, as its name indicates, is prevalent in adults, affecting 60% of individuals older than 60 years of age. 5 In addition, when the spinal deformity is associated with loss of sagittal alignment, there is a significant impact on the patient's quality of life. 4 6

Pain and disability have been correlated both to lumbar degenerative changes and spinopelvic sagittal alignment. 7 8 The analysis of the spinopelvic sagittal alignment protocol was adequately described and should be performed with full radiographs measuring angular and linear parameters. 6 Thus, several studies 6 9 have shown a correlation between these parameters and quality of life indicators, including the Oswestry Disability Index (ODI).

Recently, a grading scale for LDD was presented based on radiographic findings of DDD and the presence or absence of deformity, using full spine radiography. 10 11 This LDD grading scale demonstrated excellent intra- and interobserver reproducibility, reducing the need and costs of performing complementary exams that are more complex. 11

Considering the increasing incidence of LDD due to the increase in the average life expectancy and in the elderly population, a better understanding of the correlation between these processes and their effect on the quality of life of individuals is necessary. Therefore, the aim of the present study was to evaluate the clinical relevance of the LDD grading scale by comparing it with quality of life through the ODI.

## Materials and Methods

### Study Design and Setting

The present is a cross-sectional study with a retrospective analysis of a database of a single institution from 2019 to 2022. The study protocol was approved by the institutional Research Ethics Committee (CAAE: 39056420.7.0000.5463) and informed consent was obtained from each participant.

### Participants

**Fig. 1:**
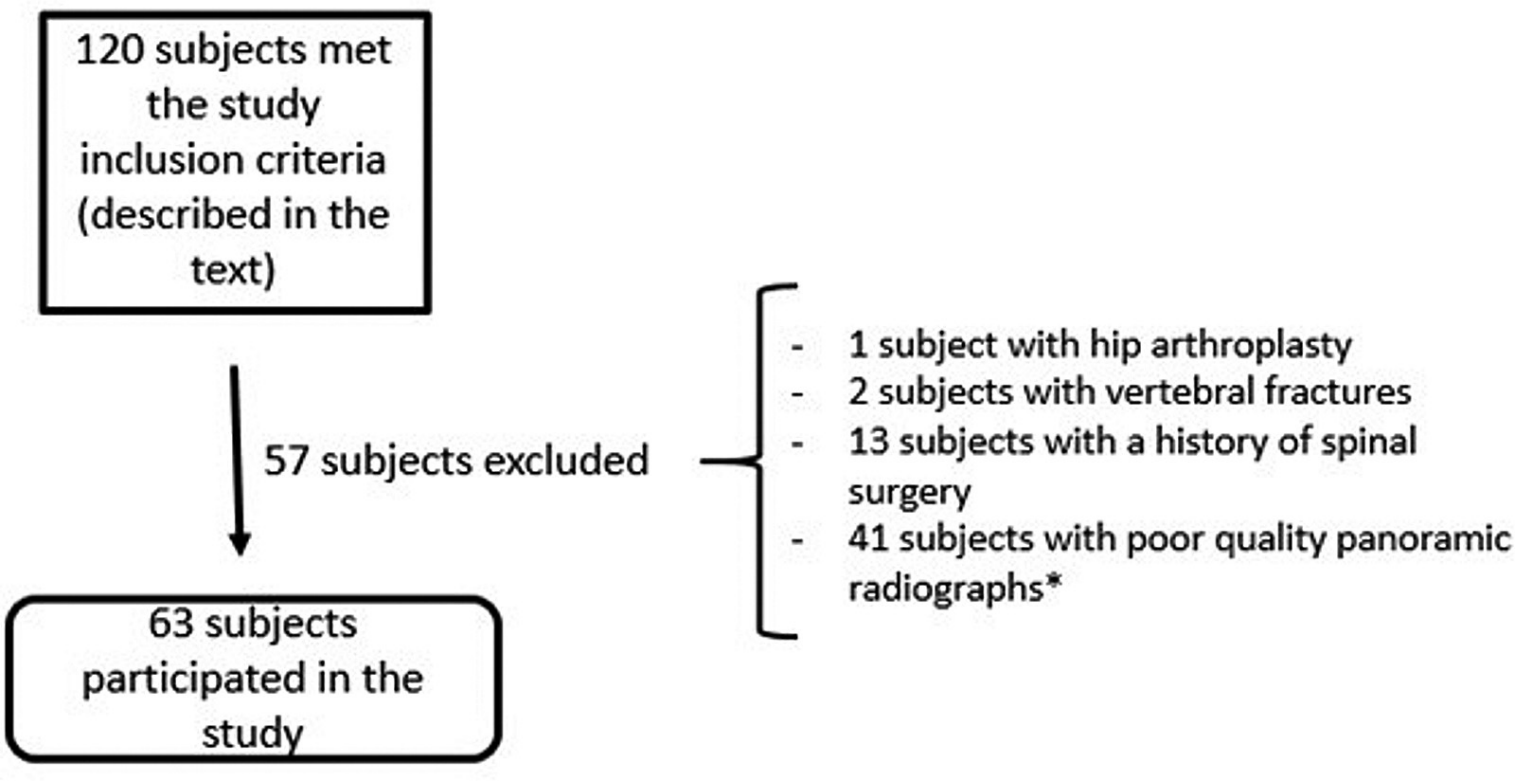
Number of patients included and excluded. **Note:** *Poor quality radiographs: those in which it was not possible to adequately visualize all the vertebrae and intervertebral spaces between the C2 vertebra and the femoral heads.

The study included patients with significant low back pain treated at the spinal disease outpatient clinic, aged over 18 years, with completed ODI questionnaires and full spine radiography. Patients with previous spinal surgery, those diagnosed with neurological or neuromuscular diseases, or with a history of spine trauma or neoplastic disease were excluded. In addition, patients whose radiographic examinations did not show a complete and appropriate view of the spine (all vertebrae and intervertebral discs visualized from the C2 vertebra to the femoral heads) were also excluded ( Chart 1 ). 12 A total of 63 patients met these criteria ( Fig. 1 ).

**Chart 1:** Technical criteria to perform a full spine X-ray

1. Proper distance from the source to the image. In general, 180 cm, as it produces acceptable enlargement and distortion of the image.
2. A compensating filter between the patient and the X-ray beam ensures adequate density between the chest cavity and the denser lumbosacral region.
3. The patient is standing, with knees extended, and feet aligned and parallel to shoulder width. Elbows and wrists flexed at the height of the supraclavicular fossa bilaterally. If there is a length discrepancy between the lower limbs of more than 2 cm, one must use compensation to align the pelvis.

### Studied Outcomes

The patient's quality of life was assessed through the ODI, 13 14 and radiographic studies ( Chart 2 ) were used to identify the presence and severity of LDD. All analyses were performed by one of the researchers, an orthopedist specializing in spine surgery. The radiographic findings determining LDD were loss of disc height or disc collapse, osteophytosis, and subchondral sclerosis, signs of instability, such as spondylolisthesis, laterolisthesis, or rotatory subluxation, in addition to the presence of scoliosis. 10 Then, the patients were classified according to the LDD radiographic grading scale into the four grades described ( Chart 2 ).

**Chart 2:** Radiographic classification of lumbar degenerative disease (LDD) 10

• Grade 0: absence of radiographic signs of degenerative disease in the lumbar spine.
• Grade I: radiographic signs of degenerative disease in one or two lumbar spine segments, without scoliosis or signs of instability.
• Grade II: radiographic signs of degenerative disease in three or more lumbar spine segments, without scoliosis or signs of instability.
• Grade III: radiographic signs of degenerative disease in the lumbar spine associated with scoliosis (coronal slope measured by the Cobb technique ≥ to 30°) and/or signs of instability, such as laterolisthesis (> 2 mm) and spondylolisthesis (at least of grade 2).

### Statistical Analysis

The statistical analysis was performed with the R (R Foundation for Statistical Computing, Vienna, Austria) software, version 2021. The Shapiro–Francia test was performed to determine the normality of the variables, and all variables presented a normal distribution. The analysis of variance (ANOVA) test was used to compare the patient's age and ODI score with the LDD grading scale. The Pearson Chi-squared test was used to compare the LDD scale in terms of sex. Values of *p* ≤ 0.05 were considered statistically significant.

The non-linear regression model was considered to evaluate the correlation of the studied variables and the ODI score (generalized additive model, GAM) to enable the analysis of the parametric variables and those with linear distribution and the non-parametric variables or those with non-linear distribution. The following variables were included: grade of LDD and age. With this, we attempted to predict the value of the patient's ODI score according to their age and LDD severity.

## Results

**Fig. 2:**
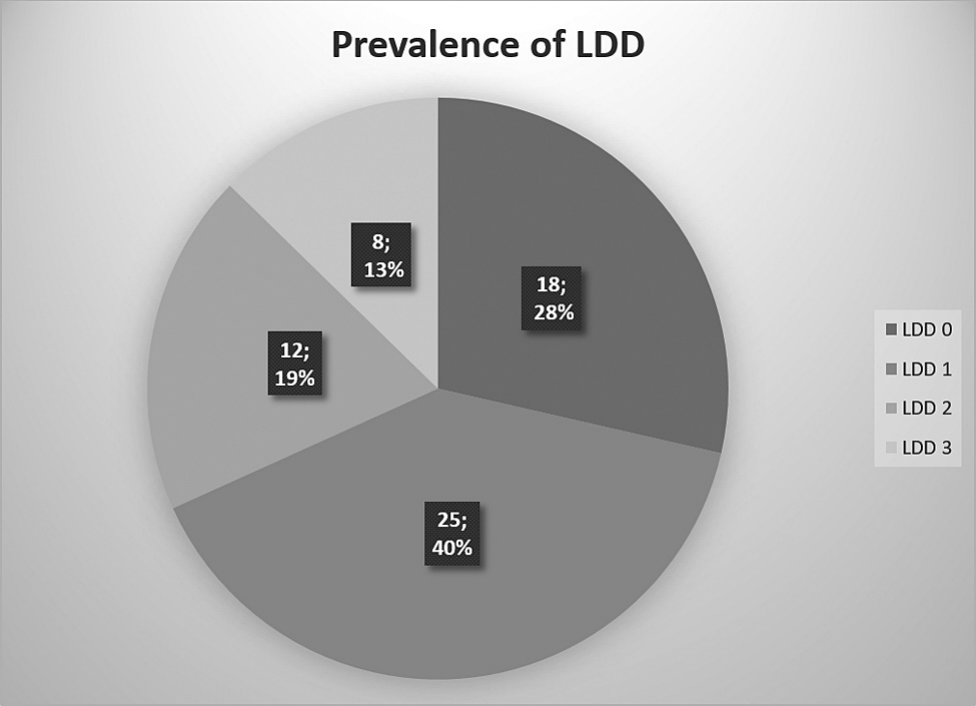
Prevalence and distribution of lumbar degenerative disease (LDD) among the study participants.

The distribution and prevalence of the study sample regarding LDD severity are shown in Fig. 2 . Most individuals were classified as grades 0 (28%) or 1 (40%) and were women (66%).

**Fig. 3:**
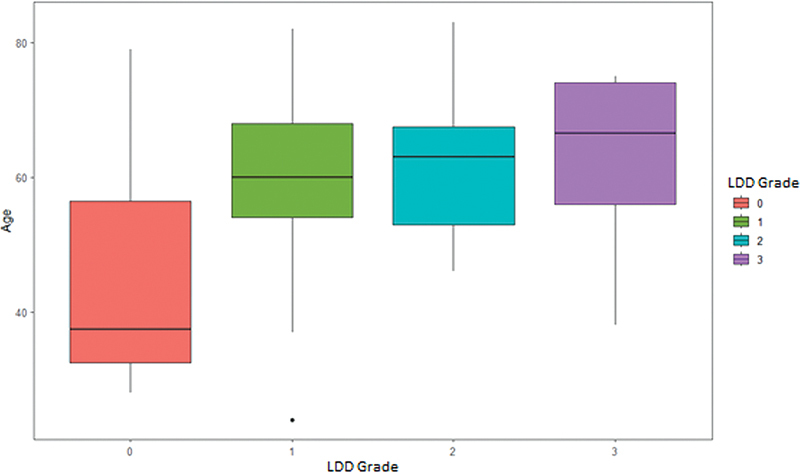
Boxplot showing the distribution by age according to the different LDD grades (analysis of variance [ANOVA]: *p* < 0.001).

Fig. 3 shows the age distribution rergarding the different grades of LDD: patients with some grade of lumbar degenerative disease (grades 1, 2, or 3) were found in the group older than 60 years of age compared with patients without lumbar degenerative disease (LDD type 0) ( *p* < 0.001). However, we did not find a statistically significant difference regarding age among the LDD groups (grades 1, 2, and 3). In addition, there was no difference regarding sex and the presence or severity of LDD, with uniformity among the grades of the scale used ( *p* = 0,81).

**Fig. 4:**
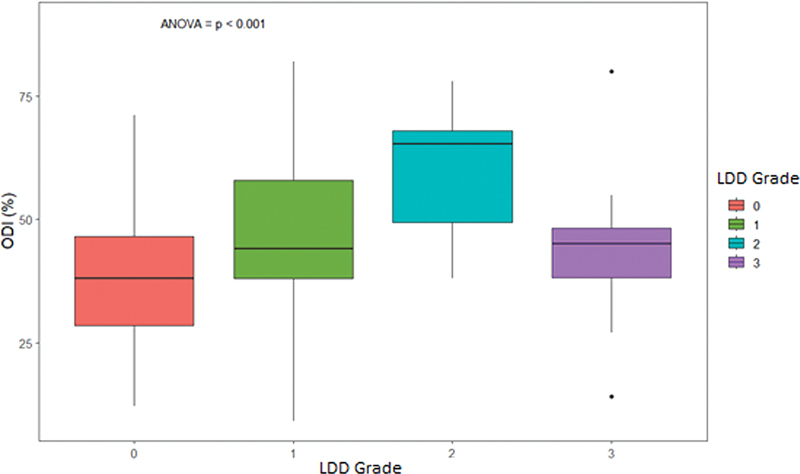
Boxplot of the relationship between quality of life (measured by the Oswestry Disability Index [ODI]) and the different LDD grades. ANOVA: *p* = 0.01 in the relationship between grades 2 and 0. No statistical significance was observed regarding the other relationships.

The relationship between quality of life (ODI score) and the LDD grading scale in our sample was statistically significant ( *p* = 0.01) when comparing LDD grade 0 and grade 2, with no statistical significance between grades 1 and 3 ( Fig. 4 ).

**Fig. 5:**
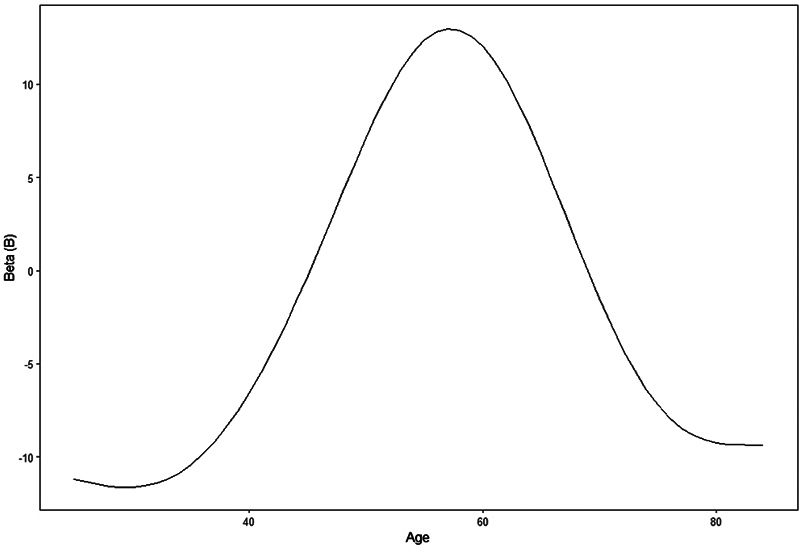
Non-linear coefficient for each age value ( *p* = 0.005).

For the development of the GAM, the following variables were included: LDD grade and age. Each LDD grade was dichotomized, with 0 indicating absence and 1, presence. Age was included as a non-linear variable ( Fig. 5 ). The general additive formula was: *Y(ODI) = intercept (a) + B1.(LDD0) + B2.(LDD1) + B3.(LDD2) + B4.(LDD3) + non-linear coefficient (age).*

Table 1 shows the value of the B coefficient for each LDD grade, remembering that each patient can only belong to one group in the LDD classification (0, 1, 2, or 3), using only one B coefficient, that of the classification to which the patient belongs. Table 1 also shows the value of the formula constant (intercept).

**Table 1:** Values of the intercept and B coefficient for each grade of lumbar degenerative disease (LDD), if present. Each patient can only belong to 1 group in the LDD classification (0, 1, 2 or 3), using only one coefficient B.

	B	95% confidence interval	*p*
Intercept	38	35–41	< 0.001
LDD 0	6.2	−1.5–14	0.12
LDD 1	6.9	1.0–13	0.026
LDD 2	17	9.2–25	< 0.001
LDD 3	7.6	−1.8–17	0.12
Age			0.005
Model evaluation			
Degrees of freedom	8		
Log-likelihood	−258		
Observations	63		
Variance explained by the model	38%		

**Fig. 6:**
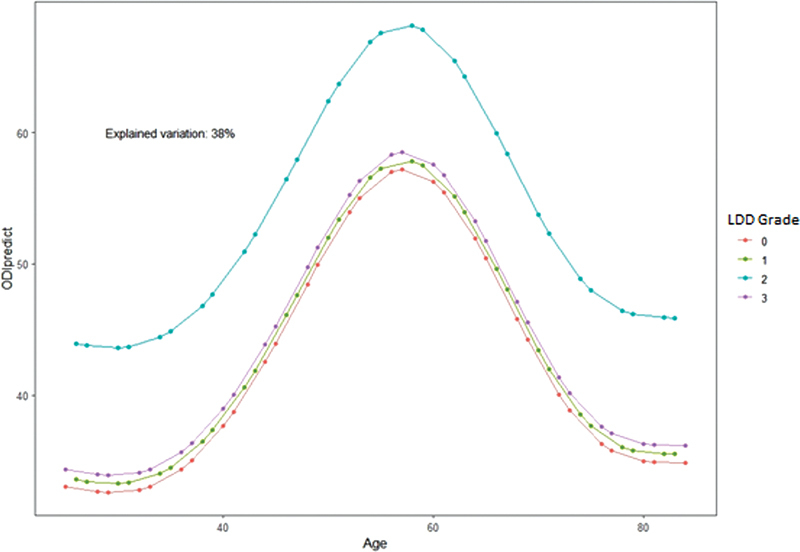
Score on the ODI predicted according to the age variation in each LDD grade, according to the sample's generalized additive model (GAM).

Fig. 6 shows the predicted value of the ODI score for quality of life in each LDD grade according to age using the GAM.

## Discussion

Although low back pain is not an exclusive symptom of LDD, these two entities are intrinsically related. 15 Corroborating these data, most study participants presented some grade of lumbar degenerative disease, with most patients classified as LDD grade 1. According to the literature, 16 most patients who seek medical care for problems related to the spine are women, with a ratio of 2:1 to men. Although there was no statistical difference regarding sex and the presence or severity of LDD, a statistical relationship was observed between age and the presence LDD. However, when evaluating each LDD grade separately, we could not find a direct relationship between the patient's age group and LDD severity according to the grading system.

The LDD grading system used in the present study has previously demonstrated good intra and interobserver reproducibility. 10 11 Hence, when correlated to the ODI score, we noticed that the LDD grading system presented a direct relationship with grades 2 and 0, and no statistical significance was found regarding the other LDD grades. Therefore, patients with LDD grade 2 present worse ODI scores than those without LDD (grade 0).

A downward trend in the ODI score (lower degree of disability) was also observed when we compared LDD grade 3 with the other grades (1 and 2), but without statistical significance. With this, it is possible to infer that DDL grade 3 is associated with any type of deformity, such as scoliosis, spondylolisthesis or laterolisthesis, regardless of the number of levels affected by degenerative characteristics, allowing to include those patients in grade 3, according to the radiographic classification considered, 11 do not have a major impact on the quality of life of these patients when compared to radiographically evident degenerative alterations of the intervertebral space without the presence of these deformities. This may occur because part of that group (LDD grade 3) are young patients with adolescent idiopathic scoliosis and some small degenerative findings in the lumbar spine, who do not suffer significant impairment in their quality of life caused by these conditions. 17 18 19 Therefore, studies with a larger number of participants are necessary to corroborate this finding, since the number of participants classified as LDD grade 3 was limited in the present study, and we were unable to obtain a statistically significant result with this comparison.

In developing the GAM formula, by which one could predict the expected ODI score according to age and LDD grade, we found a statistically significant relationship in patients with grades 1 and 2 ( Table 1 ). We observed that the LDD grade that most distanced itself from the others in Fig. 6 was grade 2, the grade that most demonstrated an impact on the ODI score compared with the others ( *p* < 0.001). Thus, this formula is another potential tool to assess the quality of life in the population with LDD, as it would enable the estimation of the ODI score according to age and radiographic LDD classification.

The present study has some limitations. First, the radiographic parameters were only analyzed by one evaluator (a spine surgeon), and the interobserver reproducibility of the classification used was not evaluated, as it had been evaluated in previous studies. 10 11 Moreover, we highlight the small size of our sample, which makes a subgroup analysis more complex. Furthermore, given the small sample size, the GAM formula, although valid, has a broader confidence interval and low predictive power (38%). Thus, studies with larger populations are needed to confirm the relationship between the radiographic grades of LDD and the clinical worsening of patients.

On the other hand, the current study showed the applicability of a new radiographic grading scale for LDD. 10 11 This classification uses a full spine radiography, a non-invasive and less expensive exam compared with other exams used to classify LDD, such as magnetic resonance imaging. 20 In addition, we analyzed this grading scale regarding its influence on the population's quality of life.

## Conclusion

The radiographic LDD grading scale is a toll of potential clinical relevance, as it presented a significant relationship in the current study with the quality of life measured by the ODI score in part of the groups evaluated.
