# Supplementary material for: Relationship between a Radiographic Grading Scale of Degenerative Lumbar Disease and Quality of Life
Source: Rev Bras Ortop (Sao Paulo). 2024 Dec 21;59(6):e861–7. doi: 10.1055/s-0044-1792115 (PMC11663057; doi:10.1055/s-0044-1792115)
Supplement: Related texts

# Research article: Relação entre uma escala radiográfica de doença degenerativa lombar e qualidade de vida

**Keywords:** degeneração do disco intervertebral, indicadores de qualidade de vida, osteoartrite da coluna vertebral, qualidade de vida relacionada com saúde, radiografia

## Abstract

**Objetivo** Avaliar a correlação entre uma escala radiográfica de doença degenerativa lombar e o Índice de Incapacidade de Oswestry (Oswestry Disability Index, ODI, em inglês).

**Métodos** Estudo transversal em que o questionário de qualidade de vida ODI e os parâmetros radiográficos para a classificação da doença degenerativa lombar em diferentes graus foram comparados para se tentar estabelecer uma relação entre eles.

**Resultados** A relação entre os parâmetros radiográficos e os indicadores de qualidade de vida não é homogênea ao se considerar os diferentes graus da escala de classificação lombar. A doença degenerativa lombar de grau 2 apresentou relação estatisticamente significativa com a piora da pontuação no ODI de qualidade de vida.

**Conclusão** A escala de classificação de doenças degenerativas lombares utilizada neste estudo demonstrou potencial clínico relevante, pois apresentou relação significativa com a qualidade de vida medida pela pontuação no ODI em parte dos grupos avaliados.

## Introdução

Uma das principais causas de dor lombar crônica é a doença degenerativa lombar (DDL), caracterizada por uma série de alterações, principalmente no disco intervertebral, denominadas doença degenerativa do disco (DDD). A evolução desse processo e os mecanismos pelos quais tais alterações causam dor ainda são temas de discussão na literatura. 1 2 Entre os achados radiográficos característicos da DDD, estão diminuição da altura do espaço discal, osteofitose e irregularidade da placa terminal, que podem ou não estar associados a deformidades como escoliose, espondilolistese ou laterolistese degenerativa. No entanto, tais achados em exames radiográficos de indivíduos assintomáticos são comuns, e a associação entre os achados descritos e a sintomatologia ainda precisa ser estabelecida por completo. 3

Outra causa importante de dor lombar crônica é o grupo de doenças que se enquadra no escopo da deformidade da coluna vertebral do adulto (DCVA), 4 que, como indica o nome, é prevalente em adultos, e acomete 60% dos indivíduos com mais de 60 anos. 5 Além disso, a associação da deformidade da coluna com a perda do alinhamento sagital tem impacto significativo na qualidade de vida do paciente. 4 6

Dor e incapacidade têm sido correlacionadas tanto a alterações degenerativas lombares quanto ao alinhamento sagital espinopélvico. 7 8 A análise do protocolo de alinhamento sagital espinopélvico foi bem descrita e deve ser realizada em radiografias completas por meio da medida de parâmetros angulares e lineares. 6 Assim, vários estudos 6 9 têm demonstrado correlação entre esses parâmetros e indicadores de qualidade de vida, inclusive o Índice de Incapacidade de Oswestry (Oswestry Disability Index, ODI, em inglês).

Recentemente, introduziu-se uma escala de classificação para DDL com base em achados radiográficos de DDD e na presença ou ausência de deformidade, usando radiografias totais da coluna vertebral. 10 11 Esta escala de classificação de DDL demonstrou excelente reprodutibilidade intra e interobservador, e reduz a necessidade e os custos de realização de exames complementares de maior complexidade. 11

Considerando a crescente incidência de DDL devido ao aumento da expectativa média de vida e da população idosa, faz-se necessário compreender melhor a correlação entre esses processos e seu efeito na qualidade de vida dos indivíduos. Portanto, o objetivo deste estudo foi avaliar a relevância clínica da escala de classificação da DDL ao compará-la com a qualidade de vida por meio do ODI.

## Materiais e Métodos

## Delineamento Experimental e Local do Estudo

Este é um estudo transversal com análise retrospectiva do banco de dados de uma instituição de 2019 a 2022. O protocolo do estudo foi aprovado pelo Comitê de Ética em Pesquisa da instituição (CAAE: 39056420.7.0000.5463), e cada participante assinou o termo de consentimento livre e esclarecido.

## Participantes

O estudo incluiu pacientes com dor lombar significativa tratados no ambulatório de doenças da coluna, com idade superior a 18 anos, questionários ODI preenchidos e radiografias totais da coluna vertebral. Pacientes com cirurgia de coluna prévia, diagnosticados com doenças neurológicas ou neuromusculares, ou com histórico de trauma de coluna ou doença neoplásica foram excluídos. Além disso, pacientes cujos exames radiográficos não permitiam a visualização completa e apropriada da coluna (todas as vértebras e discos intervertebrais da vértebra C2 até as cabeças femorais) também foram excluídos ( Quadro 1 ). 12 No total, 63 pacientes atenderam a esses critérios ( Fig. 1 ).

**Quadro 1:** Critérios técnicos para a realização de radiografia total de coluna

1. Distância adequada da fonte para a imagem: geralmente, 180 cm, pois produz ampliação e distorção aceitáveis da imagem
2. Um filtro compensador entre o paciente e o feixe de raios X garante densidade adequada entre a cavidade torácica e a região lombossacra mais densa.
3. Paciente em pé, com joelhos estendidos, pés alinhados e paralelos à largura dos ombros, e cotovelos e punhos flexionados na altura da fossa supraclavicular bilateralmente. Se houver discrepância de comprimento entre os membros inferiores maior do que de 2 cm, deve-se usar compensação para o alinhamento da pelve.

**Fig. 1:** Número de pacientes incluídos e excluídos. **Nota:** *Radiografias de baixa qualidade: aquelas que não permitiam a visualização adequada de todas as vértebras e espaços intervertebrais entre a vértebra C2 e as cabeças femorais.

## Desfechos Estudados

A qualidade de vida dos pacientes foi avaliada por meio do ODI, 13 14 e estudos radiográficos ( Quadro 2 ) foram usados para identificar a presença e a gravidade da DDL. Todas as análises foram realizadas por um dos pesquisadores, um ortopedista especialista em cirurgia de coluna. Os achados radiográficos determinantes de DDL foram perda de altura do disco ou colapso do disco, osteofitose e esclerose subcondral, sinais de instabilidade, como espondilolistese, laterolistese ou subluxação rotatória, e presença de escoliose. 10 Em seguida, os pacientes foram classificados de acordo com a escala de graduação radiográfica de DDL entre os quatro graus descritos ( Quadro 2 ).

**Quadro 2:** Classificação radiográfica da doença degenerativa lombar (DDL) 10

• Grau 0: ausência de sinais radiográficos de doença degenerativa na coluna lombar.
• Grau I: sinais radiográficos de doença degenerativa em um ou dois segmentos da coluna lombar, sem escoliose ou sinais de instabilidade.
• Grau II: sinais radiográficos de doença degenerativa em três ou mais segmentos da coluna lombar, sem escoliose ou sinais de instabilidade.
• Grau III: sinais radiográficos de doença degenerativa da coluna lombar associada à escoliose (inclinação coronal medida pela técnica de Cobb ≥ a 30°) e/ou sinais de instabilidade como laterolistese (> 2 mm) e espondilolistese (no mínimo de grau 2).

## Análise Estatística

A análise estatística foi realizada por meio do programa R (R Foundation for Statistical Computing, Viena, Áustria), versão 2021. O teste de Shapiro–Francia foi usado para se determinar a normalidade das variáveis, e todas apresentaram distribuição normal. O teste de análise de variância ( *analysis of variance* , ANOVA, em inglês) foi usado para se comparar a idade do paciente e a pontuação no ODI à escala de classificação de DDL. O teste do Qui-quadrado de Pearson foi usado para se comparar a escala de DDL entre os sexos. Valores de *p* ≤ 0,05 foram considerados estatisticamente significativos.

Por meio do modelo de regressão não linear, avaliou-se a correlação entre as variáveis estudadas e a pontuação no ODI (modelo aditivo generalizado, ou *generalized additive model* , GAM, em inglês) para permitir a análise de variáveis paramétricas e de distribuição linear e não paramétricas ou não lineares. As variáveis incluídas foram o grau de DDL e a idade. Com isso, tentou-se prever a pontuação do paciente no ODI de acordo com sua idade e gravidade da DDL.

## Resultados

A Fig. 2 mostra a distribuição e a prevalência da amostra do estudo quanto à gravidade da DDL. A maioria dos indivíduos foi classificada como de grau 0 (28%) ou 1 (40%) e composta por mulheres (66%).

**Fig. 2:** Prevalência e distribuição da doença degenerativa lombar (DDL) entre os participantes do estudo.

A Fig. 3 mostra a distribuição etária dos pacientes segundo os diferentes graus de DDL: pacientes com algum grau de DDL (1, 2 ou 3) pertenciam ao grupo acima de 60 anos em comparação a pacientes sem DDL (tipo 0) ( *p* < 0,001). No entanto, não houve diferença estatisticamente significativa em relação à idade entre os grupos com DDL de graus 1, 2 e 3. Além disso, não houve diferença em relação ao sexo e à presença ou gravidade da DDL, com uniformidade entre os graus da escala utilizada ( *p* = 0,81).

**Fig. 3:** Diagrama de caixa que mostra a distribuição por idade nos diferentes graus de DDL (análise de variância, ou *analysis of variance* , ANOVA, em inglês: *p* < 0,001).

A relação entre a qualidade de vida (pontuação no ODI) e a escala de classificação da DDL em nossa amostra foi estatisticamente significativa ( *p* = 0,01) ao se comparar os graus 0 e 2 da doença, mas não entre os graus 1 e 3 ( Fig. 4 ).

**Fig. 4:** Diagrama de caixa da relação entre qualidade de vida (medida pelo Índice de Incapacidade de Oswestry (Oswestry Disability Index, ODI, em inglês) e os diferentes graus de DDL. ANOVA: *p* = 0,01 na relação entre os graus 2 e 0. Não houve significância estatística nas demais relações.

Para a elaboração do GAM, foram incluídas as variáveis grau de DDL e idade. Cada grau de DDL foi dicotomizado, com 0 indicando ausência e 1, presença. A variável idade foi incluída como linear ( Fig. 5 ). A fórmula aditiva geral foi: *Y(ODI) = intercepto (a) + B1.(LDD0) + B2.(LDD1) + B3.(LDD2) + B4.(LDD3) + coeficiente não linear (idade).*

**Fig. 5:** Coeficiente não linear para cada valor de idade ( *p* = 0,005).

A Tabela 1 mostra o valor do coeficiente B para cada grau de DDL, lembrando que cada paciente só pode pertencer a um grupo na classificação de DDL (0, 1, 2 ou 3) e tem apenas um coeficiente B, ou seja, aquele de sua classificação. A Tabela 1 também releva o valor da constante da fórmula (intercepto).

**Tabela 1:** Valores do intercepto e do coeficiente B para cada grau de doença degenerativa lombar (DDL), caso presente. Cada paciente pode pertencer apenas a 1 grupo na classificação de DDL (0, 1, 2 ou 3), utilizando apenas um coeficiente B.

	B	Intervalo de confiança de 95%	*p*
Intercepto	38	35–41	< 0,001
DDL 0	6,2	−1,5–14	0,12
DDL 1	6,9	1,0–13	0,026
DDL 2	17	9,2–25	< 0,001
DDL 3	7,6	−1,8–17	0,12
Idade			0,005
Avaliação do modelo			
Graus de liberdade	8		
Log-verossimilhança	−258		
Observações	63		
Variância explicada pelo modelo	38%		

A Fig. 6 mostra o valor previsto da pontuação no ODI relativa à qualidade de vida em cada grau de DDL de acordo com a idade usando o GAM.

**Fig. 6:** Pontuação no ODI prevista de acordo com a variação etária em cada grau de DDL segundo o modelo aditivo generalizado ( *generalized additive model* , GAM, em inglês) da amostra.

## Discussão

Embora a dor lombar não seja um sintoma exclusivo da DDL, essas duas entidades estão intrinsecamente relacionadas. 15 Corroborando esses dados, a maioria dos participantes do estudo apresentava algum grau de DDL, em especial o grau 1. De acordo com a literatura, 16 a maioria dos pacientes que procuram atendimento médico por problemas relacionados à coluna é do sexo feminino, em proporção de 2:1 com relação aos homens. Embora não tenha havido diferença estatística em relação ao sexo com a presença ou gravidade da DDL, foi observada uma relação estatística entre a idade e a presença de DDL. No entanto, ao avaliarmos cada grau de DDL separadamente, não encontramos uma relação direta entre a faixa etária do paciente e a gravidade da DDL segundo sua classificação.

O sistema de graduação de DDL usado neste estudo já demonstrou boa reprodutibilidade intra e interobservador. 10 11 Portanto, quando correlacionado ao ODI, notamos que o sistema de graduação de DDL apresentou relação direta com os graus 2 e 0, mas não houve significância estatística entre os outros graus de DDL. Com isso, pacientes com DDL de grau 2 apresentam pontuações piores no ODI do que aqueles sem DDL (grau 0).

Também foi observada uma tendência de queda na pontuação no ODI (menor incapacidade) ao se comparar o grau 3 da DDL com os demais graus (1 e 2), mas sem significância estatística. Com isso, é possível inferir que a DDL grau 3 está associada a qualquer tipo de deformidade, como escoliose, espondilolistese ou laterolistese, independentemente do número de níveis acometidos pelas características degenerativas, permitindo incluir aqueles pacientes no grau 3, segundo a classificação radiográfica considerada, 11 Não apresentam grande impacto na qualidade de vida destes pacientes quando comparadas às alterações degenerativas do espaço intervertebral radiograficamente evidentes sem a presença destas deformidades. Isso pode ocorrer porque parte desse grupo (DDL de grau 3) são jovens com escoliose idiopática do adolescente e alguns pequenos achados degenerativos na coluna lombar que não sofrem comprometimento significativo em sua qualidade de vida por essas doenças. 17 18 19 Portanto, estudos com um número maior de participantes são necessários para corroborar esse achado, uma vez que o número de participantes com DDL de grau 3 foi limitado, e não conseguimos obter um resultado estatisticamente significativo nessa comparação.

Ao elaborarmos a fórmula GAM, que poderia prever o valor esperado da pontuação no ODI de acordo com a idade e o grau de DDL, encontramos uma relação estatisticamente significativa em pacientes com DDL de graus 1 e 2 ( Tabela 1 ). Observamos que o grau de DDL que mais se distanciou dos demais na Fig. 6 foi o DDL 2, que mais demonstrou impacto na pontuação no ODI em comparação aos demais ( *p* < 0,001). Isso torna esta fórmula mais uma possível ferramenta para o estudo da qualidade de vida na população com DDL, pois permitiria estimar a pontuação no ODI de acordo com a idade e a classificação radiográfica da DDL.

Este estudo tem algumas limitações. Primeiro, os parâmetros radiográficos foram analisados somente por um profissional (um cirurgião de coluna), e a reprodutibilidade interobservador da classificação utilizada não foi avaliada por ter sido feita em estudos anteriores. 10 11 . Além disso, destacamos o pequeno tamanho da amostra, o que torna a análise de subgrupos mais complexa. Devido ao pequeno tamanho da amostra, a fórmula GAM, embora válida, tem um intervalo de confiança mais amplo e baixo poder preditivo (38%). Assim, estudos com populações maiores são necessários para confirmar a relação entre os graus radiográficos de DDL e a piora clínica dos pacientes.

Por outro lado, nosso estudo mostrou a aplicabilidade de uma nova escala de classificação radiográfica para a DDL. 10 11 Esta classificação utiliza uma radiografia total da coluna, um exame não invasivo e menos dispendioso em comparação a outros exames utilizados para a classificação de doenças degenerativas lombares, como a ressonância magnética. 20 Além disso, analisamos essa escala de classificação quanto à sua influência na qualidade de vida da população.

## Conclusão

A escala de graduação radiográfica da DDL é uma ferramenta com possível relevância clínica, pois apresentou relação significativa neste estudo com a qualidade de vida medida pelo ODI em parte dos grupos avaliados.
